# Deep Learning in Diagnosis of Dental Anomalies and Diseases: A Systematic Review

**DOI:** 10.3390/diagnostics13152512

**Published:** 2023-07-27

**Authors:** Esra Sivari, Guler Burcu Senirkentli, Erkan Bostanci, Mehmet Serdar Guzel, Koray Acici, Tunc Asuroglu

**Affiliations:** 1 Department of Computer Engineering, Cankiri Karatekin University, Cankiri 18100, Turkey; 2 Department of Pediatric Dentistry, Baskent University, Ankara 06810, Turkey; 3 Department of Computer Engineering, Ankara University, Ankara 06830, Turkey; 4 Department of Artificial Intelligence and Data Engineering, Ankara University, Ankara 06830, Turkey; 5 Faculty of Medicine and Health Technology, Tampere University, 33720 Tampere, Finland

**Keywords:** deep learning, dental anomalies and diseases, dental diagnostics, dental images, convolutional neural network

## Abstract

Deep learning and diagnostic applications in oral and dental health have received significant attention recently. In this review, studies applying deep learning to diagnose anomalies and diseases in dental image material were systematically compiled, and their datasets, methodologies, test processes, explainable artificial intelligence methods, and findings were analyzed. Tests and results in studies involving human-artificial intelligence comparisons are discussed in detail to draw attention to the clinical importance of deep learning. In addition, the review critically evaluates the literature to guide and further develop future studies in this field. An extensive literature search was conducted for the 2019–May 2023 range using the Medline (PubMed) and Google Scholar databases to identify eligible articles, and 101 studies were shortlisted, including applications for diagnosing dental anomalies (*n* = 22) and diseases (*n* = 79) using deep learning for classification, object detection, and segmentation tasks. According to the results, the most commonly used task type was classification (*n* = 51), the most commonly used dental image material was panoramic radiographs (*n* = 55), and the most frequently used performance metric was sensitivity/recall/true positive rate (*n* = 87) and accuracy (*n* = 69). Dataset sizes ranged from 60 to 12,179 images. Although deep learning algorithms are used as individual or at least individualized architectures, standardized architectures such as pre-trained CNNs, Faster R-CNN, YOLO, and U-Net have been used in most studies. Few studies have used the explainable AI method (*n* = 22) and applied tests comparing human and artificial intelligence (*n* = 21). Deep learning is promising for better diagnosis and treatment planning in dentistry based on the high-performance results reported by the studies. For all that, their safety should be demonstrated using a more reproducible and comparable methodology, including tests with information about their clinical applicability, by defining a standard set of tests and performance metrics.

## 1. Introduction

Today, although most oral and dental diseases have early diagnosis and treatment opportunities with technological developments in oral and dental health, their global increase cannot be prevented. According to the WHO Global Oral Health Status Report (2022) [1], oral and dental diseases affect approximately 3.5 billion people worldwide. Especially in low- and middle-income countries, there are not adequate services in the field of oral and dental health due to the costs of diagnosis and treatment. As a result of this situation, it is estimated by the WHO that three out of four people in low- and middle-income countries are affected by oral and dental diseases [1]. The most common dental diseases, especially dental caries, are periodontal diseases, edentulism, oral cancer, dental anomalies, and cleft lip and palate diseases [1]. When efficient diagnosis and treatment are not provided for these diseases, it can cause various complications ranging from mild discomfort to death.

**Figure 1:**
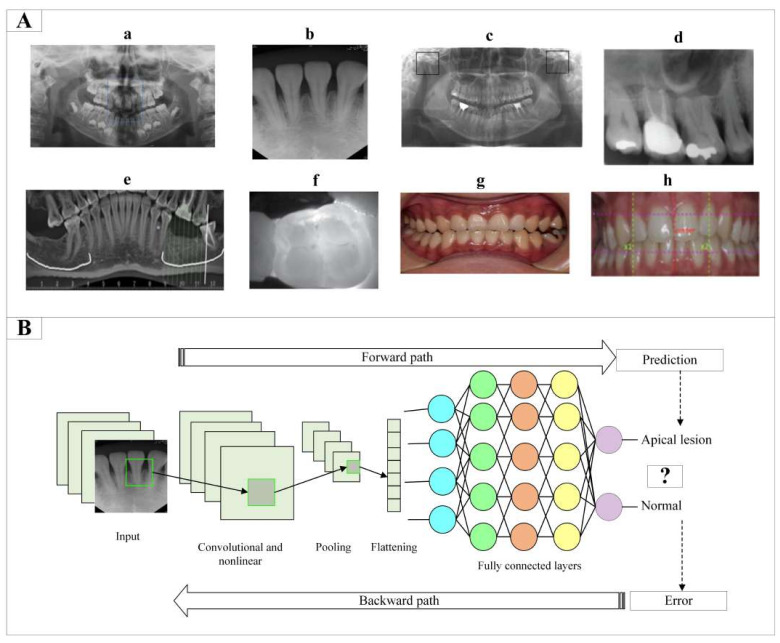
(**A**). Examples of dental anomalies and diseases on dental imaging techniques; a. Mesiodens on panoramic radiographs [21], b. Apical lesions on periapical radiographs [22], c. Temporomandibular joint osteoarthritis on orthopantomograms [23], d. Missing tooth on cone beam computed tomography [24], e. Dental caries on near-infrared-light transillumination [25], f. Dental caries on bite viewing radiographs [26], g. Dental calculus and inflammation on optical color images [27], h. Gingivitis on intraoral photos [28]. (**B**). Convolutional neural network architecture.

In addition to clinical examination, dental imaging technologies play a critical role in diagnosing oral and dental diseases. In Figure 1A, examples of some anomalies and diseases associated with dental imaging techniques are given. The advanced level of three-dimensional dental imaging technologies such as cone-beam computed tomography (CBCT), magnetic resonance imaging, and ultrasound, especially two-dimensional panoramic and periapical radiographs, has increased the success rate in diagnosis [2,3]. However, image-based dental diagnosis has some limitations. Image-based medical diagnosis is not objective as it depends on specialist experience and inter-observer variables. The background is noisy on radiographs, and anatomical structures overlap. Computed tomography has poor resolution compared to radiographs due to scattering from metallic objects. Ultrasonography contains high levels of noise. These limitations make interpreting images difficult and increase the rate of expert oversight and error.

Expert systems, aimed at assisting experts in managing images, formerly applied strict rules and methods based on how experts think. In recent years, with the ease of accessing data and the development of computers with faster processing power, artificial intelligence (AI) technologies have advanced, and expert systems have evolved into data-oriented AI applications. In particular, the increase in studies on the successful performance of deep learning methods, especially in image-based diagnostic tasks where a diagnosis is challenging, such as cancer [4], lung, and eye diseases [5,6], has increased interest in the medical application of AI [7,8]. Recent literature reviews have acknowledged the success of expert systems based on deep learning methods that compete with the performance of experts in image-based dental diagnostic tasks, especially the research presented in this article.

Deep learning is a form of machine learning that uses multilayer artificial neural networks in a wide range of applications, from image, audio, and video processing to natural language processing. Unlike traditional machine learning methods, deep learning can learn these features simultaneously by automatically extracting features from raw data symbols instead of learning with rules. In addition to these flexible structures, prediction accuracy can increase according to the size of the data. The concept of deep learning was first proposed by Hinton in 2006 as a more efficient version of multilayer artificial neural networks [9]. The CNN architecture, which is the most commonly used deep learning algorithm, is presented in Figure 1B. Since the emergence of deep learning, it has been proposed for many applications in the field of oral and dental health, such as tooth classification [10], detection [11] and segmentation [12], endodontic treatment and diagnosis [13], periodontal problem tooth detection [14], oral lesion pathology [15,16], forensic medicine applications [17,18], and classification of dental implants [19,20]. Considering the large number of images obtained in the field of oral and dental health, the dependence of dentists on computer applications in the analysis of these images, and the improvement of decision-making performance in a limited time, there seems to be excellent potential for the future of deep learning applications.

Several reviews of deep learning for oral and dental health have been published recently. These studies have focused on specific research areas such as dental caries [29], dental implants [30,31], forensic [32], endodontics [13], temporomandibular joint disorder [33], periapical radiolucent lesions [14], gingivitis and periodontal disease [34], and dental informatics [35]. Other reviews have addressed deep learning issues in dentistry [36,37,38,39,40] and dental imaging [41,42]. However, a comprehensive review study on deep learning methods used to diagnose dental diseases, including dental anomalies, has yet to be conducted. This study aims to systematically review 101 related research articles applying deep learning methods to diagnose dental anomalies and diseases.

- The essential contributions of this article can be listed as follows:
- This study is the first systematic review of dental anomalies and deep learning.
- This study includes 101 shortlisted research articles from Scholar and PubMed that apply deep learning methods for diagnosing dental anomalies and diseases.
- This review included variables such as the size of the dataset, the dental imaging method, the deep learning architecture used for performance evaluation criteria, and the explainable AI method.
- Unlike other reviews in the literature, in this review, studies comparing human-AI performance among shortlisted research articles are discussed in detail, especially statistical tests.

As per the workflow of the current article, Section 2 contains a research methodology demonstration that includes the research question, information sources, eligibility criteria, search strategy, selection process, data extraction, and analysis processes. In Section 3, the dataset features of the studies included in the shortlist were synthesized by presenting the findings, such as the deep learning method, performance metrics, and human-AI comparison. In Section 4, the findings presented in the previous section are discussed with emphasis on management implications, academic implications, literature shortcomings, and suggested solutions. The problems and suggested solutions for increasing the clinical utility of deep learning and the limitations of the current article are also included in this section. Finally, in Section 5, potential research directions are finalized. 

## 2. Material and Methods

This systematic review was conducted by referring to the PRISMA 2020 statement [43], an updated guideline for reporting systematic reviews. The review question determining the study’s eligibility criteria and search strategy is based on the PICO (problem/population, intervention/indicator, comparison, and outcome) framework in Table 1.

### 2.1. Information Sources and Eligibility Criteria

The systematic literature search was carried out by a reviewer by conducting an extensive investigation in two different electronic databases, Medline via PubMed, and Google Scholar, for studies published in the last five years (2019–May 2023). Google Scholar is a comprehensive database of scholarly material from academic research, including books, journal articles, conference reports, chapters, and theses. Google Scholar provides free services, with no subscription required. Search results are ordered by relevance, where it was published, authors, full-text match, and how often it is cited. Medline is a database containing international publications on clinical medicine and biomedical research. The PubMed database is an accessible interface service provided by Medline. The research articles included in this systematic review were selected according to the eligibility criteria below.

Inclusion criteria:

1. Articles published between January 2019–May 2023.
2. Articles on the diagnosis of dental anomalies or diseases.
3. Articles suggesting deep learning methods.
4. Articles created using a reference dataset on dental imaging techniques.
5. Full-text research articles.
6. Articles written in English.
7. The article must contain detailed information about the dataset, methods, results, and tests applied.

Exclusion criteria:

1. Articles on topics such as healthy tooth detection, tooth labeling/numbering, dental implants, and endodontic treatment.
2. Articles that have applied other AI methods that do not include deep learning methodologies, such as classical machine learning.
3. Review articles and other types such as conferences, article abstracts, book chapters, preprints, or non-full-text articles, even if it is a research article.

### 2.2. Search Strategy and Selection Process

Keywords combining techniques of interest (such as deep learning/CNN), image materials (such as radiographs), and areas of interest (such as dental anomalies/diseases) were used to navigate through articles. Medical Subject Headings (MeSH) of deep learning, CNN, convolutional neural networks, oral, dental, tooth, teeth, anomalies, and diseases were included. Included MeSH terms are combined with Boolean operators such as and/or, and advanced settings of databases are used with selections such as inclusion date range, publication types and language. The electronic search strategy applied to databases is given in Table 2.

The articles included in this systematic review were selected in two stages. In the first stage, a reviewer evaluated the articles according to the relevance of the titles and abstracts related to our research topic. In the first stage, studies with titles and abstracts unrelated to oral and dental health that could not be full-text articles, such as abstracts, were eliminated. In the second stage, a second reviewer conducted a detailed examination according to the eligibility criteria. During this examination, review articles, articles whose method was not deep learning, and articles that did not focus on oral/dental anomalies or disease diagnosis were excluded.

### 2.3. Data Extraction and Analysis

One reviewer performed the data extraction phase from the included studies. From the included articles, the primary author, publication year, anomaly/disease for which the diagnosis was intended, image type, number of images, primary performance metric and outcome value, other measured performance criteria, and explainable AI method data were obtained by reviewing detailed full texts. The shortlist presented in the article was thoroughly reviewed and checked by a second reviewer (specialist dentist). Different shortlists were made for anomaly and disease studies, and the two subjects were analyzed within themselves. The included studies were categorized as classification, object detection, and segmentation studies. Data such as the country of origin of the studies, the data division strategy determining the number of training and test datasets used in the study, and the field of dentistry were not mentioned.

The distribution of the number of publications by year, type of task, type of anomaly/disease, and dental imaging technique was visualized and analyzed. Considering the heterogeneity, performance, and outcome measures of index and reference tests for quality assessment, meta-analysis was not performed as the results were largely unsuitable for heterogeneity tests. Instead, a separate shortlist was created by selecting studies that performed tests on human-AI comparison among the included studies for quality assessment. From these studies, data on reference datasets, statistical significance tests, diagnostic performance results, diagnostic time, and the impact of AI performance were extracted and analyzed. Further analysis, including the clinical significance of deep learning, was performed narratively alongside descriptive statistics.

## 3. Results

**Figure 2:**
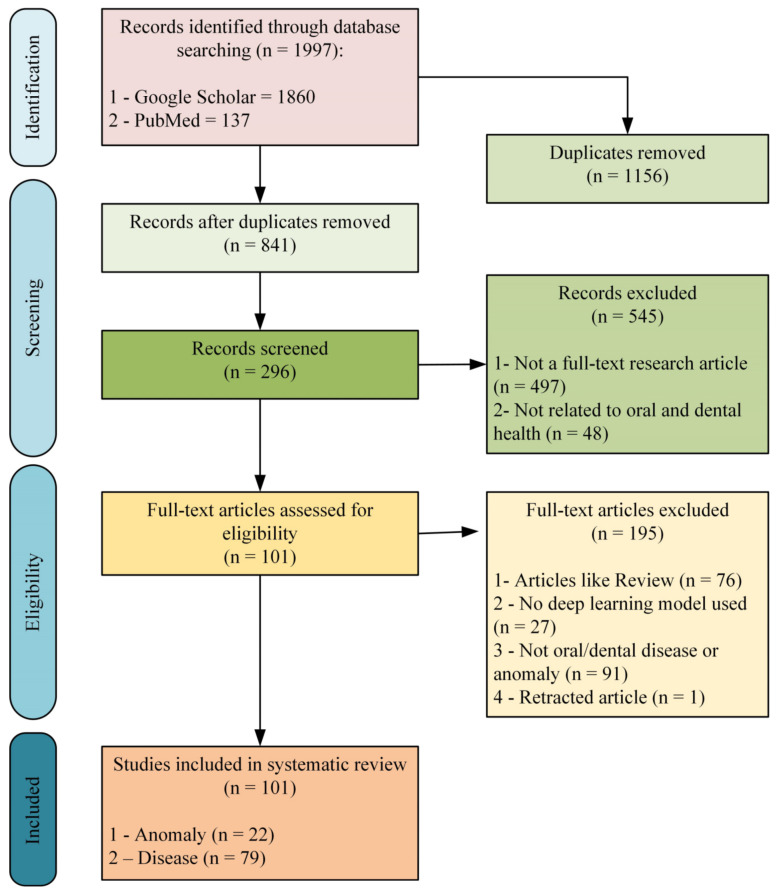
Search results according to the PRISMA-2020 flowchart.

According to the search results, a total of 1997 records were identified, including 1860 from Google Scholar and 137 from PubMed. After removing duplicates from these records, 545 studies that were not full-text research articles (*n* = 497) and not related to dental health topics (*n* = 48) were excluded, and 296 records were scanned. According to the screening results, 101 studies that met the eligibility criteria were included in the systematic review. Of the included studies, 22 are on dental anomalies (Table 3), and 79 are on dental disease (Table 4). Figure 2 presents the search results in detail according to the PRISMA-2020 flowchart.

**Figure 3:**
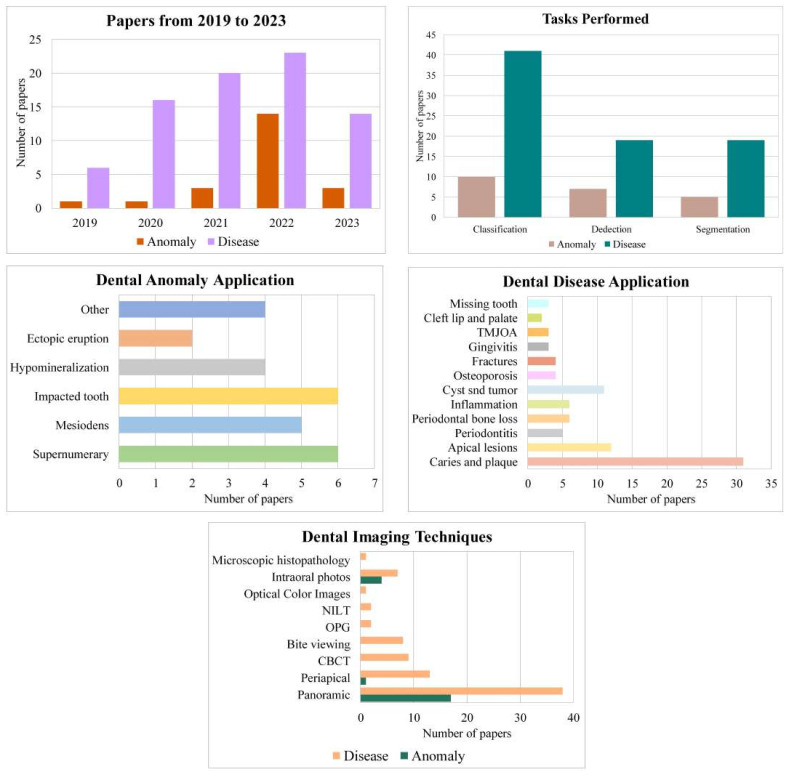
Distribution of publications by years, tasks performed, anomaly/disease applications, and dental imaging techniques.

Figure 3 shows the distribution of publications by years, tasks performed, anomaly/disease applications, and dental imaging techniques. When the distribution of the number of publications in 2019–May 2023 is examined, the highest number belongs to 2022, with 14 anomalies and 23 diseases. Although 2023 (*n* = 17) is not yet finished, the number of publications is more than double the number of publications in 2019 (*n* = 7), and the number of publications has increased yearly. The most common task performed in diagnosing dental anomaly/disease is classification (*n* = 51). Another common task performed after classification is object detection in anomaly diagnosis (*n* = 7) and segmentation in disease diagnosis (*n* = 19). The most common diagnostic studies, mainly dental caries and plaques (*n* = 31) are on periodontal diseases (*n* = 23), cysts, and tumors (*n* = 11). Cleft lip and palate (*n* = 2), temporomandibular joint osteoarthritis (TMJOA), gingivitis, and missing teeth (*n* = 3) are the least researched diseases in the diagnosis of dental disease with deep learning. Apart from these studies, there are also studies on the diagnosis of inflammation, osteoporosis, and fractures. Since mesiodens are a type of supernumerary teeth, the most common type of anomaly in which deep learning methods are used for diagnosis is supernumerary teeth (*n* = 11). Other common types of anomalies examined were impacted teeth (*n* = 6), hypomineralization (*n* = 4), and ectopic eruption (*n* = 2). Three other anomaly applications in Figure 3 are diagnosing taurodont [64], maxillary canine impaction [48], and odontomas [46]. Another study is on the classification of ten different types of anomalies [47].

Dataset sizes ranged from 60 (CBCT) [136] to 12,179 (panoramic) [87] images. The most commonly used image types in dental disease studies are panoramic radiographs (*n* = 38), followed by periapical radiographs (*n* = 13), CBCT (*n* = 9), bite viewing radiographs (*n* = 8), and intraoral photographs (*n* = 7). Orthopantomogram (OPG, *n* = 2), NILT (*n* = 2), optical color images (*n* = 1), and microscopic histopathology (*n* = 1) are other dental imaging techniques using deep learning for diagnosis. There are no studies on image types other than panoramic radiographs (*n* = 17), intraoral photographs (*n* =4), and periapical radiographs (*n* = 1) in the diagnosis of dental anomalies. One study evaluated both CBCT scans and panoramic radiographs [88], while another evaluated both periapical and panoramic radiographs [76].

The most commonly used deep learning methods for the classification task in dental anomaly diagnosis are using pre-trained CNN models by fine-tuning (transfer learning) or modifying them. InceptionResNet, VGG16, AlexNet, InceptionV3, SqueezeNet, ResNet, and DenseNet are CNN models used as solution methods. In one study [44], Hybrid graph cut segmentation was applied to separate the background and anatomy in panoramic radiography images, and then the preprocessed images were classified with CNN. YOLO (*n* = 2), Faster R-CNN (*n* = 2), DetectNet, and EfficientDetD3 models were used for object detection tasks in dental anomaly diagnosis. In one study [55], the authors designed a new DMLnet model based on the YOLOv5 architecture for automatically diagnosing mesiodens on panoramic radiographs. Generally, U-Net (*n* = 3) or modified U-Net (*n* = 1) architectures are used for the segmentation task. In a study for the diagnosis of mesiodens [60], varied tasks were performed by segmentation with the DeepLabV3 model and classification with the InceptionResNetV2 model.

While more diverse than the classification methods used for dental anomaly diagnosis, the most commonly used deep learning method is the same for disease diagnosis, with models designed with pre-trained CNNs. ResNet (*n* = 5), DenseNet (*n* = 5), and AlexNet (*n* = 4) are the most commonly used pre-trained CNNs, with VGG (*n* = 2), Inception (*n* = 2), EfficientNet (*n* = 2), LeNet (*n* = 2), and MobileNet (*n* = 1) also used. Another common method is custom CNN models designed by the authors (*n* = 6). In addition to these methods, hybrid methods combined with two different algorithms were also used. CNN-LSTM [70], CNN-SVM [71], Siamese Network-DenseNet121 [71], and CNN-fuzzy logic [84] are hybrid models using. In a study [74], a swine transformer, one of the transformer types shown to compete with CNNs recently, was used. Faster R-CNN (*n* = 7), DetectNet (*n* = 5), YOLO (*n* = 4), Single-Shot Detector (SSD, *n* = 2), and Mask R-CNN (*n* = 1) were used for the object detection task. U-Net (*n* = 14) and DeepLabV3+ (*n* = 2) were the most commonly used architectures for the segmentation task in disease diagnosis as well as in dental anomaly diagnosis. Two-stage methods combining different tasks are frequently proposed for diagnosing dental diseases. After applying segmentation as an image preprocessing in the first stage, the studies that involved classification in the second stage used U-Net + DenseNet (*n* = 2), Mask R-CNN + CNN, Morphology-based Segmentation + Modified LeNet, Curvilinear Semantic DCNN + InceptionResNetV2 methods. In a study [27], a parallel 1D CNN was used as a YOLOv5 classifier as an image preprocessing method. To optimize the weights, methods that combine CNN with different optimization algorithms, such as antlion [83] and pervasive deep gradient [26], have also been proposed.

In sixty studies, the ACC metric was used as the primary performance measurement method. While the ACC metric was measured in nine studies, it was never used in thirty-two. In a study using Faster R-CNN to diagnose gingivitis from intraoral photographs, the highest ACC value of 100% was obtained [26]. The lowest ACC value of 69% was obtained in a study using ResNet18 to diagnose dental caries on NILT images [67]. After ACC, the most frequently used performance measurement method is SEN (SEN = recall = TPR), which was used in eighty-seven studies, nine of which were the primary metric. The values obtained in studies using SEN as the primary metric range from 81–99% [59,122]. Another frequently used metric is precision (precision = PPV). Precision was used as a performance measurement method in sixty-seven studies, of which nine were the primary metric. As the lowest value, the mean average precision (mAP) value of 59.09% was reached in a study where Faster R-CNN was recommended for detecting missing teeth on panoramic radiographs [119]. The highest precision value of 98.50% was achieved in a study that proposed YOLOv4 for detecting mandibular fractures on panoramic radiographs [117]. Another performance measurement method used as a primary metric is the Area under the ROC Curve (AUC). AUC was used in thirty studies, nine of which were the primary metric, and gave results in the 57.10–99.87% range [47,91]. F score (*n* = 47) and SPEC (*n* = 48) are among other frequently used metrics. In addition, some studies use Intersection over Union (IoU), negative predictive value (NPV), Dice similarity coefficient (DSC), Jaccard similarity coefficient (JSC), Matthews correlation coefficient (MCC), false positive rate (FPR), loss, error rate (ER), and Classification rate (CR) as performance measurement methods.

Of the 101 studies, 22 mentioned the topic of explainable AI. Five of these studies described the class activation heat map (CAM) without detailing their explainable AI method, while the others used gradient-weighted class activation mapping (Grad-CAM).

In 21 studies, human and AI performances were tested and compared. The reference data, comparative tests, and performance results of these studies are summarized in Table 5. In these studies, test datasets prepared for the reference dataset were also used in comparative tests, and the size of the test datasets varied between 25 and 800 [86,87]. In one study [47], a different test dataset of 7697 images was used to test the model’s performance, and a different test dataset of 30 images was used to compare the model’s performance with the human’s performance. In one study [77], validation performance was used for tests for which no test dataset was created. In 14 studies, reference datasets were annotated by physicians experienced in oral and dental health, such as pediatric specialists (PS), general practitioners (GP), oral and maxillofacial radiologists (OMFR), surgeons (OMFS), and endodontic specialists (ES). In 10 studies, more than one specialist was given the task of explanation to ensure the reliability of the reference dataset. In two studies, dentist-trained researchers annotated the reference dataset [74,123]. In two studies, CBCT data were referenced rather than annotated, as images from retrospective databases were already labeled [23,97].

In all studies except for two, comparative tests were performed by comparing the performance results of a group of human auditors on the test data with the performance results of the model. In addition to this test, in two studies, the performance of the AI-unaided group and the AI-aided group were compared, and the effect of the AI model on the diagnostic performance of the specialists was measured [75,124]. Statistical analysis tests such as the Kruskal–Wallis test, *t*-tests, Mann–Whitney-u test, and Kappa statistics, especially McNemar’s χ^2^ test, were used to measure the significance of performance differences between specialists and AI models. Statistical significance was not measured in one study [87]. Of the eighteen studies whose *p*-value was calculated, thirteen reported that the performance difference was significant (*p* < 0.05), and five were insignificant. In addition to test performance, test times were also measured in seven studies. In only one of these studies, the AI model provided a diagnosis later than the specialist [74]. In other studies, the authors only compared the diagnostic performances, stating that the diagnostic time of the AI model would be shorter than that of the specialists. Except for six studies, the diagnostic performance of AI models proposed in other studies exceeded that of human auditory groups. In four of the six studies, AI lags behind the experts by a small margin, and in the other two studies, the performance gap is quite significant [21,47].

## 4. Discussion

This study evaluated the last five years of literature research on the diagnosis of dental anomalies and diseases using various deep learning methods, mainly CNNs, using a systematic review. According to the results of this evaluation, some findings that need to be discussed have emerged.

Even if it is not as much as the diagnostic applications in the field of medicine, the results of the searches made in two databases, Google Scholar, and PubMed, show the records of 1997 for the last five years, which shows that deep learning is experiencing a golden age in the diagnostic applications in dentistry. Only 137 of these records were available on PubMed, which is often an essential medical and dental research resource. This case indicates that a significant part of the research identified is not from dentistry but from technical sciences and has been published differently.

Over the years, deep learning has grown in popularity as a research topic in diagnosing dental diseases, given the number of studies shortlisted before the first half of 2023. However, diagnosing dental anomalies with deep learning has yet to be sufficiently investigated (*n* = 22). This article is the first systematic review of dental anomalies. Due to the rarity of dental anomalies compared to other dental diseases, the scarcity of data has made research on this subject with deep learning algorithms rare [137,138]. In addition, it is no coincidence that the most common oral and dental disease reported worldwide by the WHO is dental caries and that the studies included in the shortlist are primarily focused on diagnosing dental caries (*n* = 31). Due to the working principle of deep learning algorithms, the qualities of the data used, such as the number of images, quality, and an expert’s explanation of the image, are very important compared to other AI algorithms. These findings prove that deep learning has gained more space in the literature for diagnosing common worldwide diseases where it is easy to obtain quality data. In general, the progress of the health sector in the world draws the boundaries of the field of AI in medicine. Similar to this subject, panoramic radiographs are the most widely used imaging technique in the field of oral and dental health worldwide due to their advantages over others, and panoramic radiographs were used as data in more than half (*n* = 55) of the studies included in the shortlist in this review.

Since classification is the most appropriate task type for disease diagnosis in general, the most classification (*n* = 51) and the least segmentation (*n* = 24) task types were performed in the studies included in the shortlist. Despite the fact that deep learning algorithms can work with raw data, segmentation and object detection are used as preprocessing tools applied to the data before classification in order to overcome the difficulties of dental images. Although they are used as individual or at least individualized architectures as deep learning algorithms, standardized architectures such as pre-trained CNN models, Faster R-CNN, YOLO, and U-Net have been used in most studies. Considering the existence of these architectures in the literature, it shows that deep learning and diagnostic studies in dentistry lag behind other fields. The use of transformer architectures in only one study, a relatively new field of research according to CNNs, indicates a possible delay in adopting the latest architectures. Explainable AI methods are used to explain the decision-making processes of models. Visualizing why and how deep learning models, defined as black box AI models, make the diagnosis decision is vital to making the model’s accuracy, objectivity, and results reliable. Of the 101 included studies, only 22 mentioned an explainable AI method (Grad-CAM). Considering the clinical importance of deep learning diagnostic studies, it is essential to include explainable AI methods in studies for reliability. In addition, developing new and different explainable AI methods is very important.

Although the high performance of the proposed deep learning algorithms indicates their reliability, appropriate metrics were not selected for their performance in the clinical setting, and additional tests were not carried out. In some studies, only ACC was used as a performance metric [27,65,68,80,123,136]. ACC class imbalance can be misleading in existing problems. Likewise, the AUC is only partially informative when over- or under-detection is unimportant. In problems involving such inequalities, additional metrics must be measured. Although PPV = precision, one of the metrics giving information about the clinical benefit, was measured in 67 studies, NPV was measured in only 16 studies. In addition to the limitations of the reported metrics, the number of studies applying tests that provide information on clinical utility is very small. The number of studies comparing human and AI is 21. In some of these studies, the number of explanatory experts forming the reference data set is one [21,47,133]; in others, the researcher is used instead of an expert as an explanatory [74,123]. Using more experts to overcome the limitations of a single expert in creating reference datasets will increase reliability. In some studies, performance measurements obtained with validation data have been reported instead of creating a separate test dataset [77]. The validation phase is used to test the efficiency of the hyperparameters of the deep learning model, and its use in the final testing phase may make the reported results misleading. Only two studies test how deep learning affects expert performance in AI and human benchmark tests (AI-unaided group-AI-aided group comparison) [75,124]. In other studies, deep learning algorithms and the performance of experts were compared, and it was hoped that deep learning performance would reach or exceed that of experts. At this point, although the aim of using expert system applications supported by AI algorithms as an auxiliary tool for experts is emphasized in almost all studies, it is clear that tests suitable for this purpose need to be revised. As a result of this inadequacy, it will take many years for research on the clinical applicability and ethical and legal dimensions of AI algorithms to multiply. As every multidisciplinary task requires, the cooperation of health institutions and experts with computer scientists is the most critical factor in preventing this situation. Another vital solution factor may be defining a standard set of tests and performance criteria for deep learning oral and dental health studies. An open-access, standardized test dataset created by experts for each dental image type can enable the performance of deep learning algorithms to be reliably evaluated and compared.

This systematic review article has some limitations. Since today’s databases and publications are quite large, only two different databases were scanned for this review. The selected articles were evaluated in line with the inclusion and exclusion criteria and the boundaries drawn. Studies such as conferences, preprint articles, and book chapters were excluded because the inclusion criteria were broad. The fact that some articles are not open access or contain missing information that does not match the summary tables we have created has limited the included studies. One reviewer performed the data extraction phase from the included studies, and the shortlist presented in the article was only thoroughly reviewed and checked by a second reviewer (a specialist dentist). A traditional systematic review was used; meta-analyses were not conducted, and the results were quite broad. As a result, the study findings were compiled narratively and according to a systematization we designed, with the aim of guiding and further developing future studies in this field.

## 5. Conclusions

In this systematic review, deep learning diagnosis of dental anomalies and diseases was discussed, and 101 studies included in the shortlist were analyzed and evaluated with the limitations discussed. Deep learning algorithms show auspicious performance in evaluating visual data for diagnosing dental anomalies and diseases. Applications of deep learning in oral and dental health services can alleviate the workload of oral and dental health professionals by allowing more comprehensive, reliable, and objectively accurate image evaluation and disease detection, and can increase the chance of developing countries reaching diagnosis and treatment by reducing the cost. In order to achieve these advantages of deep learning, there seems to be a great need for the development of clinical applications of deep learning studies in the field of oral and dental health, including the definition of standard test datasets, testing procedures, and performance metrics.

## Figures and Tables

**Table 1:** Definition of the research question within the framework of PICO.

Research Question	What Are the Applications and Performance of Deep Learning for Diagnosing Dental Anomalies and Diseases?
Population	Diagnostic medical images of patients with dental anomalies or disease (radiographs, CBCT, intraoral images, near-infrared-light transillumination (NILT) images, optical color Images, microscopic histopathology)
Intervention	Deep learning-based models for diagnosis and clinical decision making
Comparison	Expert diagnosis
Outcome	Predicted results that can be measured with performance metrics (accuracy (ACC), sensitivity (SEN), specificity (SPEC), Area Under the Curve (AUC), Matthews Correlation Coefficient (MCC), Intersection over Union (IoU), Positive/Negative Predictive Values (PPV/NPV), etc.)

**Table 2:** Structure of electronic search strategies.

Database	Search Strategy	Search Date
Google Scholar	all: (“deep learning” OR “CNN” OR “convolutional neural network”) AND (“oral” OR “dental” OR “tooth” OR “teeth”) AND (“anomalies” OR “diseases”)	26 May 2023
Medline/PubMed	(“deep learning”[All Fields] OR “CNN”[All Fields] OR “convolutional neural network”[All Fields]) AND (“oral”[All Fields] OR “dental”[All Fields] OR “tooth” [All Fields] OR “teeth” [All Fields]) AND (“anomalies” [All Fields] OR “diseases” [All Fields])	25 May 2023

**Table 3:** Summary of studies on deep learning diagnosis of dental anomalies.

Author, Year, Reference	Anomaly	Image Type	Dataset Size	Method	Primary Performance Metrics and Values (%)	Other Performance Metrics	Explainable AI Method
Classification							
Ahn et al., 2021, [21]	Mesiodens	Panoramic	1100	InceptionResNetV2	ACC: 92.40	Precision, Recall, F1 score, AUC	Grad-CAM
Kheraif et al., 2019, [44]	Supernumerary, Number teeth, Jaws position, Structure, Restoration, Implants, Cavities	Panoramic	1500	Hybrid Graph Cut Segmentation + CNN	ACC: 97.07	Precision, Recall, F1 score, SPEC	-
Mine et al., 2022, [45]	Supernumerary	Panoramic	220	VGG16	ACC: 84.00	SEN, SPEC, AUC	-
Okazaki et al., 2022, [46]	Supernumerary, Odontomas	Panoramic	150	AlexNet	ACC: 70.00	Precision, SEN, F1 score	-
Ragodos et al., 2022, [47]	Supernumerary, Rotation, Agenesis, Mammalons, Microdontia, Impacted, Hypoplasia, Incisal Fissure, Hypocalcification, Displaced	Intraoral photos	38,486	ResNet18	AUC for supernumerary class: 57.10	Precision, Recall, F1 score	Grad-CAM
Aljabri et al., 2022, [48]	Maxillary canine impaction	Panoramic	416	InceptionV3	ACC: 92.59	Precision, Recall, F1 score, SPEC	Grad-CAM
Liu et al., 2022, [49]	Ectopic eruption of maxillary first molars	Panoramic	1580	CNN-based Fusion Model	SPEC: 86	SEN, F1 score, PPV, NPV	Grad-CAM
Askar et al., 2021, [50]	White spot lesions, Hypomineralized lesions	Intraoral photos	434	SqueezeNet	ACC: 84.00	SEN, SPEC, F1 score, AUC, PPV, NPV	Grad-CAM
Schönewolf et al., 2022, [51]	Molar-incisor-hypomineralization, Enamel breakdown	Intraoral photos	3241	ResNeXt-101	ACC: 95.20	SEN, SPEC, AUC, PPV, NPV	Grad-CAM
Alevizakos et al., 2022, [52]	Molar-incisor-hypomineralization, Amelogenesis imperfecta, Dental fluorosis, White spot lesions	Intraoral photos	462	DenseNet121	ACC: 92.86	Loss	-
Detection							
Ha et al., 2021, [53]	Mesiodens	Panoramic	612	YOLOv3	ACC: 96.20	SEN, SPEC	-
Jeon et al., 2022, [54]	Mesiodens	Periapical	720	EfficientDetD3	ACC: 99.20	SEN, SPEC	-
Dai et al., 2023, [55]	Mesiodens	Panoramic	850	Authors Specific CNN: DMLnet	ACC: 94.00	SEN, SPEC, mAP	-
Kuwada et al., 2020, [56]	Supernumerary	Panoramic	550	DetectNet	AUC: 96.00	Precision, Recall, F1 score, ACC	-
Celik, 2022, [57]	Third molar impacted teeth	Panoramic	440	YOLOv3	mAP: 96.00	IoU, ACC, Precision, Recall	-
Başaran et al., 2022, [58]	Impacted tooth, Residual root, and eight fine-grained dental anomalies	Panoramic	1084	Faster R-CNN InceptionV2 (COCO)	SEN for Impacted class: 96.58	TP, FP, FN, Precision, F1 score	-
Lee et al., 2022, [59]	Supernumerary, Impacted, Residual root, and 14 fine-grained dental anomalies	Panoramic	23,000	Faster R-CNN	SEN: 99.00	Precision, SPEC	-
Segmentation							
Kim et al., 2022, [60]	Mesiodens	Panoramic	988	DeepLabV3plus + InceptionResNetV2	ACC: DeepLabV3plus +: 83.90, InceptionResNetV2: 97.10	IoU, MeanBF score, Precision, Recall, F1 score	Grad-CAM
Ariji et al., 2022, [61]	Third molar impacted teeth	Panoramic	3200	U-Net	DSC: 83.10	JSC, SEN	-
Imak et al., 2023, [62]	Impacted tooth	Panoramic	304	Authors Specific CNN: ResMIBCU-Net: an encoder–decoder network with residual blocks, modified inverted residual block, and bi-directional ConvLSTM	ACC: 99.82	IoU, Recall, F1 score	-
Zhu et al., 2022, [63]	Ectopic eruption of first permanent molars	Panoramic	285	nnU-Net	ACC: 99.00	DSC, IoU, Precision, SEN, SPEC, F1 score	-
Duman et al., 2023, [64]	Taurodont	Panoramic	434	U-Net	SEN: 86.50	TP, FP, FN, Precision, F1 score	-

ACC, Accuracy; AUC, Area Under the ROC Curve; CAM, Class Activation Mapping; CBCT, Cone Beam Computed Tomography; CNN, Convolutional Neural Network; DSC, Dice Similarity Coefficient; FN, False Negative; FP, False Positive; Grad-CAM, Gradient-weighted Class Activation Mapping; IoU, Intersection over Union; JSC, Jaccard Similarity Coefficient; mAP, mean Average Precision; NILT, Near-Infrared-Light Transillumination; NPV, Negative Predictive Value; OPG, Or-thopantomogram; PPV, Positive Predictive Value; SEN, Sensitivity; SPEC, Specificity; TMJOA, Temporomandibular Joint Osteoarthritis; TP, True Positive; YOLO, You Only Look Once.

**Table 4:** Summary of studies on deep learning diagnosis of dental diseases.

Author, Year, Reference	Disease	Image Type	Dataset Size	Method	Primary Performance Metrics and Values (%)	Other Performance Metrics	Explainable AI Method
Classification							
Megalan Leo and Kalpalatha Reddy, 2020, [65]	Dental caries	Bite viewing	480	InceptionV3	ACC: 86.70	-	-
Wang et al., 2020, [66]	Dental caries, Dental plaque	Intraoral photos	7200	Authors Specific CNN	ACC: Dental caries: 95.30, Dental plaque: 95.90	SEN, SPEC	-
Schwendicke et al., 2020, [67]	Dental caries	NILT	226	ResNet18	ACC: 69.00	SEN, SPEC, AUC, PPV, NPV	CAM
Megalan Leo and Kalpalatha Reddy, 2021, [68]	Dental caries: Enamel, Dentin, Pulp, Root lesions	Bite viewing	480	Hybrid Neural Network (HNN)	ACC: 96.00	-	-
Vinayahalingam et al., 2021, [69]	Dental caries	Panoramic	400	MobileNetV2	ACC: 87.00	SEN, SPEC, AUC	CAM
Singh and Sehgal, 2021, [70]	G.V Black dental caries	Periapical	1500	CNN-LSTM	ACC: 96.00	Precision, SEN, SPEC, F1 score, G-mean, AUC	-
Bui et al., 2022, [71]	Dental caries	Panoramic	95	Pretrained CNNs-SVM	ACC: 93.58	SEN, SPEC, F1 score, PPV, NPV	-
Vimalarani and Ramachandraiah, 2022, [26]	Dental caries	Bite viewing	1000	Pervasive deep gradient-based LeNet	ACC: 98.74	SEN, SPEC, ER, PPV, NPV	-
Panyarak et al., 2023, [72]	Dental caries	Bite viewing	2758	ResNet152	ACC: 71.11	SEN, SPEC, CR, AUC	CAM
Haghanifar et al., 2023, [73]	Dental caries	Panoramic	470	Authors Specific CNN: PaXNet: Ensemble transfer learning and capsule classifier	ACC: 86.05	Loss, Precision, Recall, F0.5 score	Grad-CAM
Zhou et al., 2023, [74]	Dental caries	Panoramic	304	Swin Transformer	ACC: 85.57	Precision, Recall, F1 score	-
Ezhov et al., 2021, [75]	Dental caries, Periapical lesion, Periodontal bone loss	CBCT	1346	U-Net + DenseNet	SEN: 92.39	SPEC	-
Rajee and Mythili, 2021, [76]	Dental caries, Periapical infection, Periodontal, and Pericoronal diseases	Periapical, Panoramic	2000	Curvilinear Semantic DCNN+ InceptionResNetV2	ACC: 94.51	MCC, DSC, JSC, ER, Precision, Recall, SPEC	-
Pauwels et al., 2021, [77]	Periapical lesion	Periapical	280	Authors Specific CNN	SEN: 87.00	SPEC, AUC	-
Calazans et al., 2022, [78]	Periapical lesion	CBCT	1000	Siamese Network + DenseNet121	ACC: 70.00	SPEC, Precision, Recall, F1 score	-
Sankaran, 2022, [79]	Periapical lesion	Panoramic	1500	Improved Multipath Residual CNN (IMRCNN)	ACC: 98.90	SEN, SPEC, Precision, F1 score	-
Li et al., 2021, [22]	Dental apical lesions	Periapical	476	Authors Specific CNN	ACC: 92.50	Loss	-
Chuo et al., 2022, [80]	Dental apical lesions	Periapical	760	AlexNet	ACC: 96.21	-	-
Li et al., 2022, [81]	Dental caries, Periapical periodontitis	Periapical	4129	Modified ResNet18	F1 score: Dental caries: 82.90, Periapical periodontitis: 82.80	SEN, SPEC, AUC, PPV, NPV	Grad-CAM
Liu et al., 2022, [82]	Dental caries, Periapical periodontitis, Periapical cysts	Periapical	1880	DenseNet121	ACC: 99.50	SEN, SPEC, PPV, NPV	CAM
Park et al., 2023, [27]	Calculus and Inflammation	Optical Color Images	220	YOLOv5 + Parallel 1D CNN	ACC: 74.54	-	-
Jaiswal and Bhirud, 2023, [83]	Erosive wear, Periodontitis	OPG	500	CNN with Antlion Optimization	ACC: 77.00	Precision, Recall, F1 score	-
Chauhan et al., 2023, [84]	Dental pulpitis	Periapical	428	CNN-Fuzzy logic	ACC: 94.00	SEN, SPEC, Precision, F1 score, MCC	Grad-CAM
Chang et al., 2020, [85]	Periodontal bone loss, Periodontitis	Panoramic	330	Mask R-CNN + CNN	Pixel ACC: 92.00	DSC, JSC	-
Krois et al., 2019, [86]	Periodontal bone loss	Panoramic	85	Authors Specific CNN	ACC: 81.00	SEN, SPEC, F1 score, AUC, PPV, NPV	-
Kim et al., 2019, [87]	Periodontal bone loss	Panoramic	12,179	Authors Specific CNN: DeNTNet: Deep Neural Transfer Network	F1 score: 75.00	Precision, Recall, AUC, NPV	Grad-CAM
Lee et al., 2020, [88]	Odontogenic cyst	Panoramic, CBCT	Panoramic 1140, CBCT 986	InceptionV3	AUC: Panoramic: 84.70, CBCT: 91.40	SEN, SPEC	-
Rao et al., 2021, [89]	Odontogenic cysts	Microscopic histopathology	2657	DenseNet169	ACC: 93.00	Loss, Precision, Recall, F1 score	-
Sivasundaram and Pandian, 2021, [90]	Dental cyst	Panoramic	1171	Morphology-based Segmentation + Modified LeNet	ACC: 98.50	CR, Precision, F1 score, DSC, SEN, SPEC, PPV, NPV	-
Lee et al., 2019, [91]	Osteoporosis	Panoramic	1268	Multicolumn DCNN	AUC: 99.87	ACC, Precision, Recall, F1 score	-
Lee et al., 2020, [92]	Osteoporosis	Panoramic	680	VGG16	AUC: 85.80	SEN, SPEC, ACC	Grad-CAM
Sukegawa et al., 2022, [93]	Osteoporosis	Panoramic	778	EfcientNet Ensemble Model	ACC: 84.50	Precision, Recall, F1 score, AUC	Grad-CAM
Tassoker et al., 2022, [94]	Osteoporosis	Panoramic	1488	AlexNet	ACC: 81.14	SEN, SPEC, F1 score, AUC	Grad-CAM
Nishiyama et al., 2021, [95]	Mandibular condyle fractures	Panoramic	400	AlexNet	ACC: 84.50	SEN, SPEC, AUC	-
Yang et al., 2023, [96]	Vertical root fractures	CBCT	1641	ResNet50	AUC: 92.90	SEN, SPEC, ACC, PPV, NPV	CAM
Murata et al., 2019, [97]	Maxillary sinusitis	Panoramic	920	AlexNet	ACC: 87.50	SEN, SPEC, AUC,	-
Li et al., 2021, [98]	Gingivitis	Intraoral photos	625	CNN with Multi-task Learning	AUC: 87.11	SEN, SPEC, FPR,	Grad-CAM
Choi et al., 2021, [23]	TMJOA	OPG	1189	ResNet	ACC: 80.00	SEN, SPEC, Cohen’s Kappa	-
Jung et al., 2023, [99]	TMJOA	Panoramic	858	EfficientNetB7	ACC: 88.37	SEN, SPEC, AUC	Grad-CAM
Kuwada et al., 2022, [100]	Cleft palate	Panoramic	491	DetectNet, VGG16	AUC: DetectNe: 95.00, VGG16: 93.00	SEN, SPEC, ACC	-
Al-Sarem et al., 2022, [101]	Missing tooth	CBCT	500	U-Net + DenseNet169	Precision: 94.00	ACC, Recall, F1 score, Loss, MCC	-
Detection							
Zhang et al., 2022, [102]	Dental caries	Intraoral photos	3932	Single-Shot Detector	AUC: 95.00	TPR	-
Chen et al., 2021, [103]	Dental caries, Periapical periodontitis, Periodontitis	Periapical	2900	Faster R-CNN	IoU: Dental caries: 71.59, Periapical periodontitis: 69.42, Periodontitis: 68.35	AP, AUC, Recall	-
Kim et al., 2022, [104]	Dental caries, Periapical radiolucency, Residual root	Panoramic	10,000	Fast R-CNN	ACC: 90.00	SEN, SPEC, Precision	-
Chen et al., 2022, [105]	Dental caries	Bite viewing	978	Faster R-CNN	ACC: 87.00	SEN, SPEC, PPV, NPV	-
Park et al., 2022, [106]	Dental caries	Intraoral photos	2348	Faster R-CNN	ACC: 81.30	AUC, SEN, AP	-
Fatima et al., 2023, [107]	Periapical lesions	Periapical	534	Lightweight Mask R-CNN	ACC: 94.00	IoU, mAP	-
Jiang et al., 2022, [108]	Periodontal bone loss	Panoramic	640	U-Net + YOLOv4	ACC: 77.00	AP, Recall, F1 score	-
Thanathornwong and Suebnukarn, 2020, [109]	Periodontally compromised teeth	Panoramic	100	Faster R-CNN	Precision: 81.00	SEN, SPEC, F1 score	-
Kwon et al., 2020, [110]	Odontogenic cysts	Panoramic	1282	YOLOv3	ACC: 91.30	SEN, SPEC, AUC	-
Yang et al., 2020, [111]	Odontogenic cysts	Panoramic	1603	YOLOv2	Precision: 70.70	Recall, ACC, F1 score	-
Ariji et al., 2019, [112]	Radiolucent lesions in the mandible (Ameloblastomas, Odontogenic keratocysts, Dentigerous cysts, Radicular cysts, Simple bone cysts)	Panoramic	285	DetectNet	SEN: 88.00	IoU, FPR	-
Kise et al., 2023, [113]	Mandibular radiolucent cyst-like lesions (Radicular cyst, Dentigerous cyst, Odontogenic keratocyst, Ameloblastoma)	Panoramic	310	DetectNet	ACC: 89.00	SEN, SPEC	-
Kuwana et al., 2020, [114]	Inflamed maxillary sinuses, Maxillary sinus cysts	Panoramic	611	DetectNet	ACC: 92.00	SEN, SPEC, FPR	-
Watanabe et al., 2021, [115]	Maxillary cyst-like lesions	Panoramic	410	DetectNet	Precision: 90.00	Recall, F1 score	-
Fukuda et al., 2020, [116]	Vertical root fractures	Panoramic	300	DetectNet	Precision: 93.00	Recall, F1 score	-
Son et al., 2021, [117]	Mandibular Fractures	Panoramic	420	YOLOv4	Precision: 98.50	Recall, F1 score,	-
Alalharith et al., 2020, [28]	Gingivitis	Intraoral photos	134	Faster R-CNN	ACC: 100	Recall, mAP	-
Lee et al., 2020, [118]	TMJOA	CBCT	3514	Single-Shot Detector	ACC: 86.00	Precision, Recall, F1 score	-
Park et al., 2022, [119]	Missing tooth	Panoramic	455	Faster R-CNN	mAP: 59.09	AP, IoU	-
Segmentation							
Casalegno et al., 2019, [25]	Dental caries	NILT	217	U-Net	mIoU: 72.70	AUC	-
Khan et al., 2021, [120]	Dental caries, Alveolar bone recession, Interradicular radiolucencies	Periapical	206	U-Net	mIoU: 40.20	DSC, Precision, Recall, NPV, F1 score	-
Cantu et al., 2020, [121]	Dental caries	Bite viewing	3686	U-Net	ACC: 80.00	SEN, SPEC, PPV, NPV, MCC, F1 Score	-
Bayrakdar et al., 2022, [122]	Dental caries	Bite viewing	621	U-Net	SEN: 81.00	Precision, F1 score	-
You et al., 2020, [123]	Dental plaque	Intraoral photos	886	DeepLabV3+	mIOU: 72.60	-	-
Lee et al., 2021, [124]	Dental caries	Bite viewing	304	U-Net	Precision: 63.29	Recall, F1 score, PPV	-
Lian et al., 2021, [125]	Dental caries	Panoramic	1160	Caries detection: nnU-Net, Caries severity detection: DenseNet121	IoU: nnU-Net: 78.50, ACC: DenseNet121: 95.70	DSC, Precision, Recall, NPV, F1 score	-
Zhu et al., 2022, [126]	Dental caries	Panoramic	1159	Authors Specific CNN: CariesNet	DSC: 93.64	ACC, Precision, Recall, F1 score	-
Ari et al., 2022, [127]	Dental caries, Periapical lesion	Periapical	1169	U-Net	SEN: Dental caries: 82.00, Periapical lesion: 92.00	Precision, F1 Score	-
Dayı et al., 2023, [128]	Dental caries	Panoramic	504	Authors Specific CNN: DCDNet	F1 score: 61.86	Precision, Recall, c	-
Rajee and Mythili, 2023, [129]	Dental caries	Panoramic	2000	Curvilinear Semantic DCNN	ACC: 93.7	DSC, JSC, TPR, FPR	-
Kirnbauer et al., 2022, [130]	Periapical lesion	CBCT	144	U-Net	ACC: 97.30	SEN, SPEC, FNR, DSC	-
Song et al., 2022, [131]	Dental apical lesions	Panoramic	1000	U-Net	IoU: 82.80	Precision, Recall, F1 score	-
Chen et al., 2023, [132]	Periodontal bone loss	Periapical	8000	U-Net-based Ensemble Model	ACC: 97.00	AP	-
Endres et al., 2020, [133]	Periapical inflammation, Granuloma, Cysts, Osteomyelitis, Tumor	Panoramic	2902	U-Net	PPV: 67.00	TPR, AP, F1 score	-
Yu et al., 2022, [134]	Odontogenic cysts	Panoramic	10,872	MoCoV2 + U-Net	ACC: MoCoV2: 88.72, IoU: U-Net: 70.84	Precision, F1 score, SEN, SPEC	Grad-CAM
Chau et al., 2023, [135]	Gingivitis	Intraoral photos	567	DeepLabV3plus	SEN: 92.00	SPEC, IoU	-
Wang et al., 2021, [136]	Cleft lip and palate	CBCT	60	3D U-Net	DSC: 77.00	-	-
Bayrakdar et al., 2021, [24]	Missing tooth	CBCT	75	3D U-Net	Right percentages: 95.30	False percentages	-

ACC, Accuracy; AP, Average Precision; AUC, Area Under the ROC Curve; CAM, Class Activation Mapping; CBCT, Cone Beam Computed Tomography; CNN, Convolutional Neural Network; CR, Classification Rate; DSC, Dice Similarity Coefficient; ER, Error Rate; FN, False Negative; FNR, False Negative Rate; FP, False Positive; FPR, False Positive Rate; Grad-CAM, Gradient-weighted Class Activation Mapping; IoU, Intersection over Union; JSC, Jaccard Similarity Coefficient; LSTM, Long Short-Term Memory; mAP: mean Average Precision; MCC, Matthews Correlation Coefficient; mIoU, mean Intersection over Union; NILT, Near-Infrared-Light Transillumination; NPV, Negative Predictive Value; OPG, Orthopantomogram; PPV, Positive Predictive Value; R-CNN, Region-based Convolutional Neural Network; SEN, Sensitivity; SPEC, Specificity; SSD, Single Shot Detector; SVM, Support Vector Machine; TMJOA, Temporomandibular Joint Osteoarthritis; TN, True Negative; TNR, True Negative Rate; TP, True Positive; TPR, True Positive Rate; YOLO, You Only Look Once.

**Table 5:** Summary of studies involving human-AI comparisons.

Author, Year, Reference	Test Dataset	Reference Dataset Annotators	Comparator Dentists	Statistical Significance Test	Diagnostic Performance (%)	Diagnostic Time	AI Performance (+) Effective, (−) Noneffective
Ahn et al., 2021, [21]	Panoramic, 100	1 PS	6 GP, 6 PS	Kruskal–Wallis test, *p* < 0.05	(ACC) GP: 95.00, PS: 99.00, AI Model: 88.00	GP: 811.8 s, PS: 375.5 s, AI Model: 1.5 s	Performance: −, Time: +
Ragodos et al., 2022, [47]	Intraoral photos, Reference test size 7.697, Comparative test size 30	1 SD	1 SD	Pre-calibration performance measurements	(F1 score for mammalons class) SD: 85.70, AI Model: 50.60	SD: 1 year, AI Model: 16 min for the entire dataset	Performance: −, Time: +
Liu et al., 2022, [49]	Panoramic, 100	3 PS	3 PS	Cochran test, *p* = 0.114	(SPEC) PS1: 88.00, PS2: 83.00, PS3: 87.00, AI Model: 86.00	PS: -, AI Model: 1 s	Performance: −, Time: +
Zhu et al., 2022, [63]	Panoramic, 65	1 OMFR, 2 PS	2 GP, 1 PS	McNemar’s χ^2^ test, *p* < 0.001	(ACC) GP1: 82.50, GP2: 83.30, PS: 77.50, AI Model: 99.00	-	Performance: +
Zhou et al., 2023, [74]	Panoramic, 30	An experienced data annotation worker trained by dentists	2 SD	Kappa statistic	(ACC) SD: 88.42, AI Model: 85.57	SD: 64.5 s, AI Model: 68.97 s	Performance: − Time: −
Ezhov et al., 2021, [75]	CBCT, 600	OMFR	4 OMFR	Student’s *t*-test, *p* < 0.05	(SEN) OMFR1: 94.11, OMFR2: 94.38, OMFR3: 93.18, OMFR4: 93.37, AI Model: 92.39	-	Performance: −
	CBCT, 40	OMFR	12 AI-aided group, 12 AI-unaided group	Mann–Whitney-u test, *p* < 0.05	(SEN) AI-unaided group: 76.72, AI-aided group: 85.37	AI-unaided group: 18.74 min, AI-aided group: 17.55 min	Performance: +, Time: +
Pauwels et al., 2021, [77]	Periapical, 112 (Val. dataset)	3 OMFR	3 OMFR	Quadratic weighted kappa	(SEN) OMFR: 58.00, AI Model: 87.00	-	Performance: +
Li et al., 2022, [81]	Periapical, 300	3 SD	3 JD	Kappa statistic	(F1 score) JD1: 61.29, JD2: 61.87, JD3: 65.39, AI Model: 82.85	-	Performance: +
Chang et al., 2020, [85]	Panoramic, 34	OMFR	3 OMFR (1 Professor, 1 Fellow, 1 Resident)	Intraclass Correlation Coefficient (ICC), *p* < 0.01	(ICC) AI Model-Professor: 86.00, AI Model-Fellow: 84.00, AI Model-Resident: 82.00	-	Performance: +
Krois et al., 2019, [86]	Panoramic, 25	3 SD	6 SD (1 PS, 1 ES, 4 GP)	Welch’s *t*-test, *p* = 0.067	(ACC) SD average: 76.00, AI Model: 81.00	-	Performance: +
Kim et al., 2019, [87]	Panoramic, 800	5 SD	Same 5 SD	-	(F1 score) SD average: 69.00, AI Model: 75.00	-	Performance: +
Murata et al., 2019, [97]	Panoramic, 120	CBCT	2 OMFR, 2 JD	McNemar’s χ^2^ test, *p* < 0.05	(ACC) OMFR: 89.60, JD: 76.7, AI Model: 87.50	OMFR, JD: -, AI Model: 9 s	Performance: +, Time: +
Choi et al., 2021, [23]	OPG, 450	CBCT	1 SD	McNemar’s test, *p* < 0.05	(ACC) SD: 81.00, AI Model: 80.00	-	Performance: −
Kuwada et al., 2022, [100]	Panoramic, 60	-	2 OMFR	McNemar’s χ^2^ test, *p* < 0.05	(AUC) OMFR1: 70.00, OMFR2: 63.00, AI Models: 95.00, 93.00	-	Performance: +
Chen et al., 2022, [105]	Bite viewing, 160	2 ES, 1 OMFR	2 JD	McNemar’s χ^2^ test, *p* < 0.05	(ACC) JD: 82.00, AI Model: 87.00	-	Performance: +
Yang et al., 2020, [111]	Panoramic, 181	-	3 OMFS, 2 GP	Kruskal–Wallis test, *p* = 0.77	(Precision) OMFS: 67.10, GP: 65.80, AI Model: 70.70	OMFS and GP average time: 33.8 min, AI Model: -	Performance: +, Time: +
Cantu et al., 2020, [121]	Bite viewing, 141	3 SD	7 SD	Two-sided paired *t*-test, *p* < 0.05	(ACC) SD average: 71.00, Model: 80.00	-	Performance: +
You et al., 2020, [123]	Intraoral photos, 98	A researcher	1 PS	Paired *t*-test, *p* > 0.05	(mIOU) PS: 69.50, AI Model: 73.60	-	Performance: +
	Intraoral photos, 102	A researcher	1 PS	Paired *t*-test, *p* > 0.05	(mIOU) PS: 65.20, AI Model: 72.40	-	Performance: +
Lee et al., 2021, [124]	Bite viewing, 50	2 SD	3 SD	Generalized estimating equations, *p* < 0.05	(SEN) AI-unaided group: SD1: 85.34, SD2: 85.86, SD3: 69.11, AI-aided group: SD1: 92.15, SD2: 93.72, SD3: 79.06, AI Model: 83.25	-	Performance: +
Lian et al., 2021, [125]	Panoramic, 89	4 SD	6 SD	McNemar’s χ^2^ test, *p* < 0.05	Segmentation (IoU) SD average: 69.60, AI Model: 78.50; Classification (ACC) SD average: 91.50, AI Model: 95.70	-	Performance: +
Endres et al., 2020, [133]	Panoramic, 102	1 OMFS	24 OMFS	Wilcoxon signed-rank test, *p* < 0.05	(PPV) OMFS average: 69.00, AI Model: 67.00	-	Performance: + (The AI model outperformed 14 of the 24 OMFS)

Accuracy; AUC, Area Under the ROC Curve; CBCT, Cone Beam Computed Tomography; ES, Endodontic Specialist; GP, General Practitioner; ICC, Intraclass Correlation; JD, Junior Dentist; OMFR, Oral and Maxillofacial Radiologist; OMFS, Oral and Maxillofacial Surgeon; OPG, Orthopantomogram; PPV, Positive Predictive Value; PS, Pediatric Specialist; SD, Specialist Dentist; SEN, Sensitivity; SPEC, Specificity.

## Data Availability

Not applicable.

## Abbreviations

The following abbreviations are used in this manuscript: AI, Artificial Intelligence; ACC, Accuracy; AP, Average Precision; AUC, Area Under the ROC Curve; CAM, Class Activation Mapping; CBCT, Cone Beam Computed Tomography; CNN, Convolutional Neural Network; CR, Classification Rate; DSC, Dice Similarity Coefficient; ER, Error Rate; ES, Endodontic Specialist; FN, False Negative; FNR, False Negative Rate; FP, False Positive; FPR, False Positive Rate; GP, General Practitioner; Grad-CAM, Gradient-weighted Class Activation Mapping; ICC, Intraclass Correlation; IoU, Intersection over Union; JD, Junior Dentist; JSC, Jaccard Similarity Coefficient; LSTM, Long Short-Term Memory; mAP: mean Average Precision; MCC, Matthews Correlation Coefficient; mIoU: mean Intersection over Union; NILT, Near-Infrared-Light Transillumination; NPV, Negative Predictive Value; OMFR, Oral and Maxillofacial Radiologist; OMFS, Oral and Maxillofacial Surgeon; OPG, Orthopantomogram; PPV, Positive Predictive Value; PS, Pediatric Specialist; R-CNN, Region-based Convolutional Neural Network; SD, Specialist Dentist; SEN, Sensitivity; SPEC, Specificity; SSD, Single Shot Detector; SVM, Support Vector Machine; TMJOA, Temporomandibular Joint Osteoarthritis; TN, True Negative; TNR, True Negative Rate; TP, True Positive; TPR, True Positive Rate; YOLO, You Only Look Once.
